# A fast imaging method for the interpretation of self-potential data with application to geothermal systems and mineral investigation

**DOI:** 10.1038/s41598-023-39672-8

**Published:** 2023-08-20

**Authors:** Salah A. Mehanee, Khalid S. Essa, Khaled S. Soliman, Zein E. Diab

**Affiliations:** 1 Department of Geophysics, Faculty of Science, Cairo University, Giza, 12613 Egypt; 2 School of Earth Sciences, The University of Adeliede, Adelaide, SA Australia

**Keywords:** Geophysics, Solid Earth sciences

## Abstract

We describe a rapid imaging approach for the interpretation of self-potential data collected along profile by some geometrically simple model of cylinders and spheres. The approach calculates the correlation coefficient between the analytic signal (AS) of the observed self-potential measurements and the AS of the self-potential signature of the idealized model. The depth, electric dipole moment, polarization angle, and center are the inverse parameters we aim to extract from the imaging approach for the interpretative model, and they pertain to the highest value of the correlation coefficient. The approach is demonstrated on noise-free numerical experiments, and reproduced the true model parameters. The accuracy and stability of the proposed approach are examined on numerical experiments contaminated with realistic noise levels and regional fields prior to the interpretation of real data. Following that, five real field examples from geothermal systems and mineral exploration have been successfully analyzed. The results agree well with the published research.

## Introduction

Fox^1^ proposed the self-potential method using a copper electrode and galvanometer instrument to explore a copper-sulfide ore body at Cornwall, England. The self-potential method has advanced^2–15^ and been widely used for graphite, sulfide, magnetite, uranium and gold prospecting^16–22^, mapping paleo-shear zones^23,24^, archaeological investigations^25^, geotechnical engineering^26^, cave discovery^27^, coal fires detection^28–30^, and monitor water movement^31–33^. The electrical self-potential method has been applied to a broad range of monitoring studies, like landslides or mass movements that happened by cumulative pore pressure in the rock^34^.

Self-potential is a passive technique to measure self-potential differences that occur naturally in the earth’s subsurface^14^. The mechanism and origin of the self-potential anomalies have been discussed by several authors^9,10,13,14,35–39^. Self-potential methods are preferred over other geophysical techniques in measurements that are sensitive to fluid movements through fractured and porous rock, and under natural or an applied hydraulic gradient responding to weak fluid movements^38,40^. Several approaches for self-potential data forward modeling, inversion and interpretation have been developed^10,13,41–53^. These approaches can be grouped into two categories.

**Figure 1:**
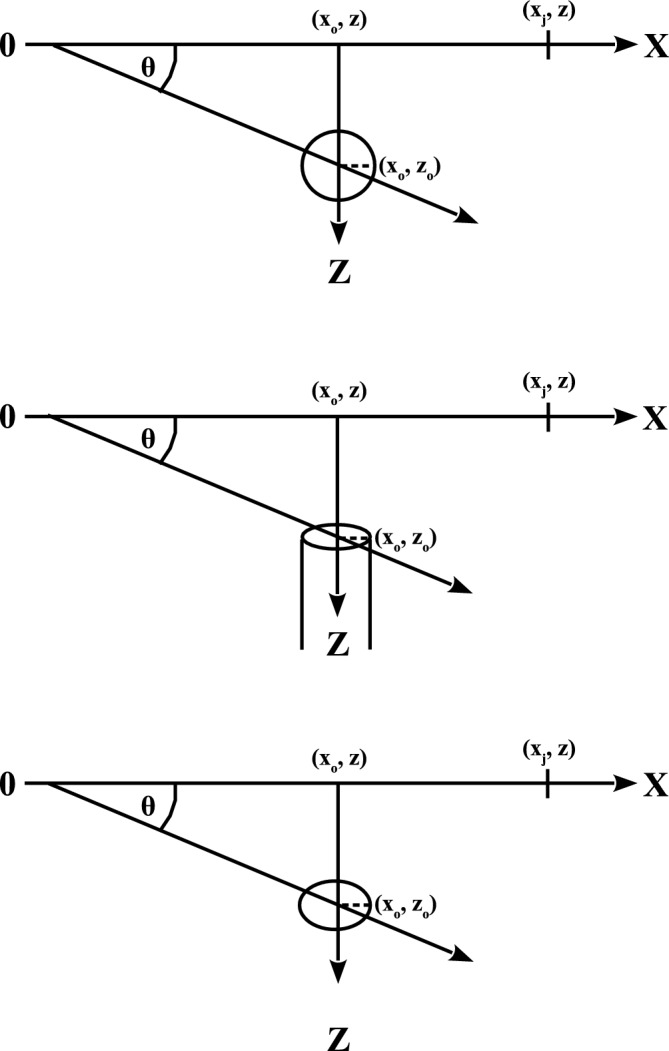
Geometry and parameters of the assumed source models. Top, middle and bottom panels present the sphere, semi-infinite vertical cylinder, and infinitely long horizontal cylinder models.

**Figure 2:**
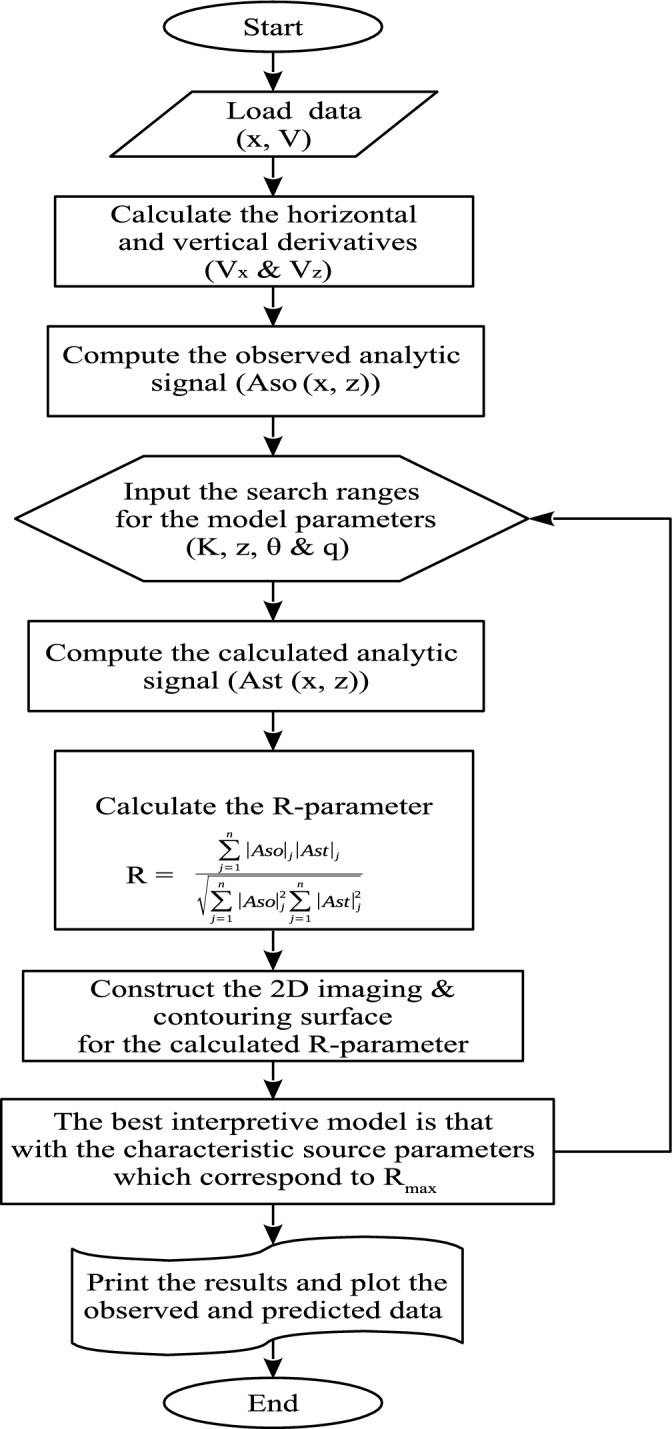
Flowchart showing the workflow of the developed scheme.

Class I is pertinent to the self-potential anomaly of multi-dimensional arbitrary structures including both the two (2D) and three-dimensional (3D) SP modeling and inversion. Multi-dimensional SP inversions can be non-unique, unstable and require large computational time^54–60^. Standard methods to retrieve a stable solution of an ill-posed inverse problem are the regularization techniques^61^.

**Table 1:** Model 1: horizontal cylinder. Shape index (q) and its corresponding maximum value of the R-parameter.

q	R-max
0.5	0.9566
0.6	0.9775
0.7	0.9912
0.8	0.9968
0.9	0.9989
**1.0**	**1.0000**
1.1	0.9991
1.2	0.9994
1.3	0.9985
1.4	0.9992
1.5	0.9985

Class II approximates the collected self-potential anomaly by some geometrically simple models, such as vertical cylinders, horizontal cylinders and spheres. This class offers a fast quantitative interpretation, and the objective is to infer the depth, location and polarization parameter of the interpretive model that best fits the observed data. The study we pursue here belongs to this class. Numerous quantitative methods (graphical and numerical) were established for Class II in order to obtain the shape, depth and polarization parameter of the causative source from the measured self-potential anomaly^43–46,49,51,53,62–65^. The disadvantage of these methods is that they are subjective, and consequently can result in some error in the model parameters^66^.

**Figure 3:**
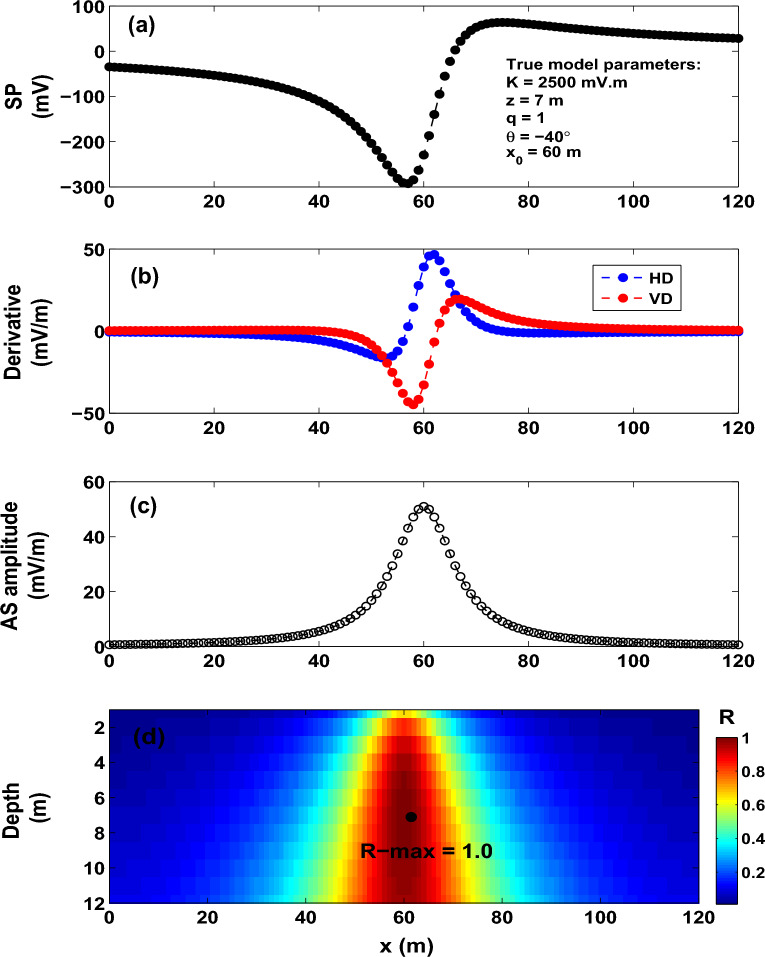
Model 1: SP data (noise free). (**a**) SP anomaly produced from a horizontal cylinder model. (**b**) Horizontal (HD) and vertical (VD) derivatives of the SP data rendered in (**a**). (**c**) Analytic signal (AS) amplitude. (**d**) Image of the R-parameter (*R*).

**Figure 4:**
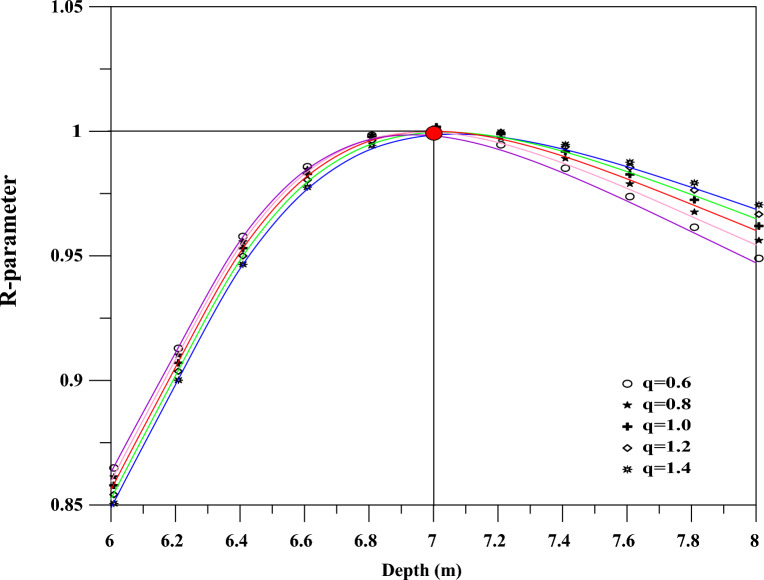
Model 1: noise-free data. Relationship of the R-parameter, shape factor, and depth.

Abdelrahman et al.^63^ introduced a graphical technique to infer the depth and shape of the buried structures from the second moving average residual self-potential anomalies. Santos^67^ applied the particle swarm optimization (PSO) scheme to invert the self-potential anomalies by some ideal geometric structures, like spheres, cylinders and inclined sheets. Mehanee^66^ developed a regularized scheme for the interpretation of self-potential data using the conjugate gradient minimizer in the space of logarithmed and non-logarithmed model parameters. Di Maio et al.^68^ presented a spectral analysis method for the interpretation of self-potential data by some geometrically simple model based upon the periodogram method (PM), multi Taper method (MTM), and maximum entropy method (MEM) to recover the depth of the anomalous body. Sungkono and Warnana^69^ applied the black hole algorithm (BHA) to self-potential data considering simple geometric bodies of sphere, horizontal cylinder and inclined sheet to determine the corresponding model parameters. The analytic signal (AS) amplitude of Nabighian^70^ can provide a key role in self-potential anomaly interpretation, and uses the spatial derivatives of the data, e.g.^71–73^. It is noted that the analytic signal method has been discussed numerously in the published literature for the interpretation of gravity and magnetic data, e.g.^71,72,74–82^.

An imaging methodology is presented in this paper for the interpretation of self-potential data by some idealized bodies (spheres and cylinders). The goal is to retrieve the self-potential profile’s origin point as well as the depth, angle of polarization, and shape index of the anomalous body. The technique estimates the correlation coefficient (the R-parameter) between the AS of the observed self-potential data and the AS of the numerical self-potential response of an assumed elucidative model. The favored elucidative model is that which attains the maximum correlation coefficient.

**Figure 5:**
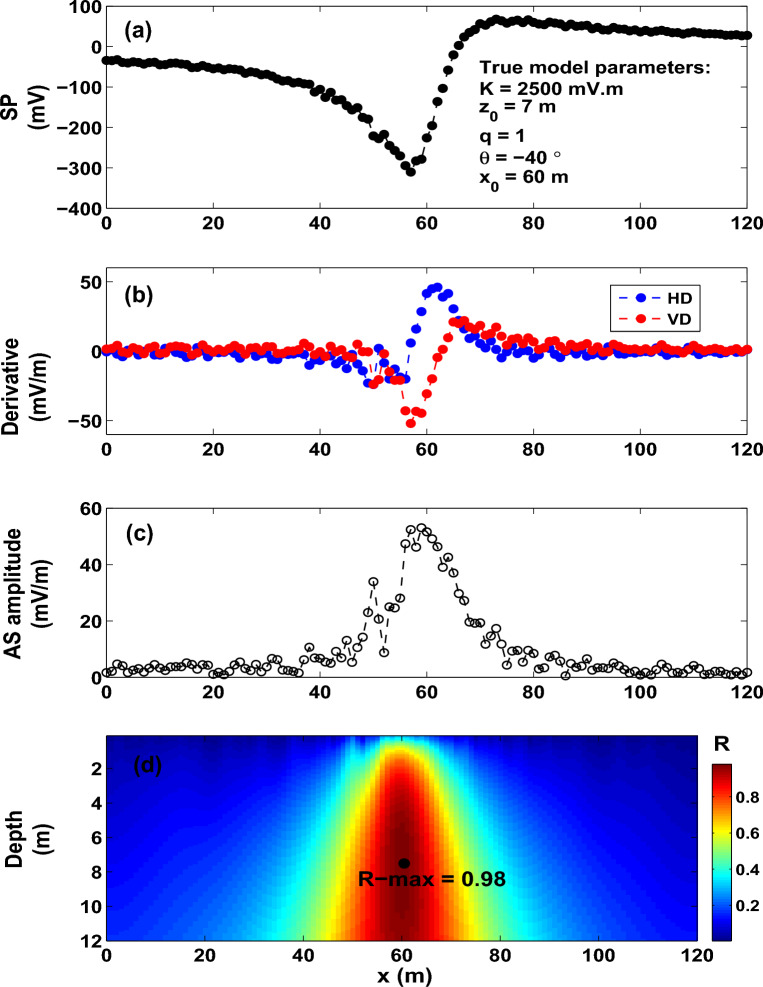
Model 2: Noisy data. (**a**) Noisy SP anomaly subject to interpretation. (**b**) Derivatives. (**c**) Amplitude. (**d**) Image.

**Figure 6:**
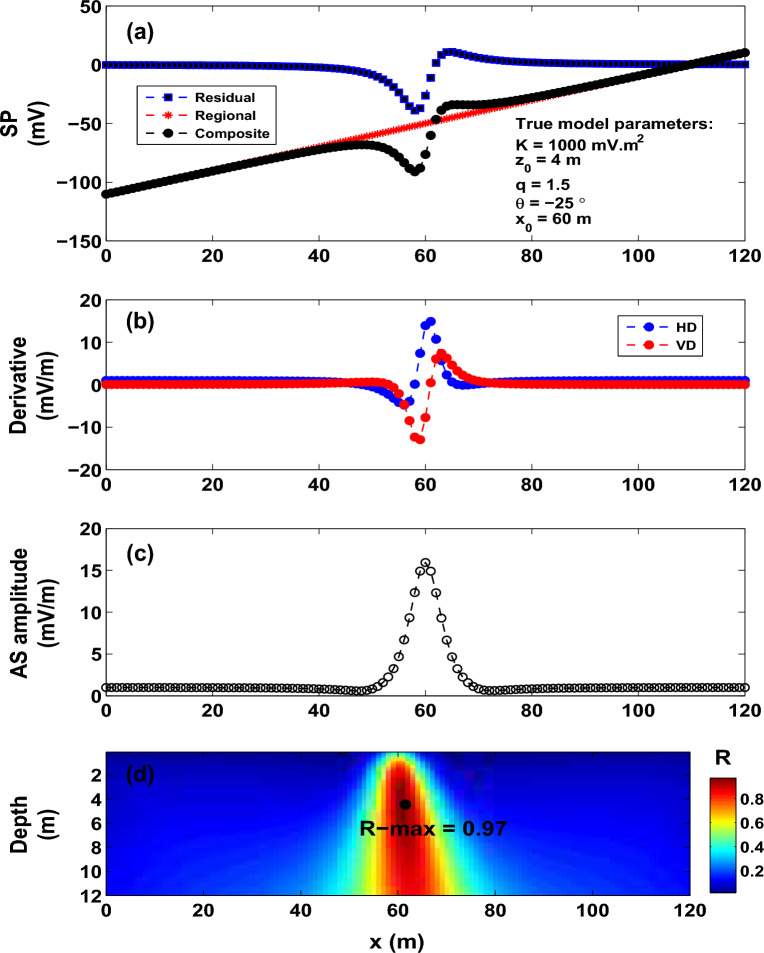
Model 3: regional anomaly’s impact. (**a**) Composite SP anomaly of sphere model and first-order regional calculated by expression (6). (**b**) Derivatives. (**c**) Amplitude. (**d**) Image.

The self-potential imaging scheme presented here retrieves the horizontal location of the anomalous body as well as its depth, polarization angle and amplitude coefficient, and has three main benefits. First, the whole self-potential data profile is used when estimating the spatial parameters (horizontal location and depth) of the buried source, which are considered key information in geophysical prospecting. Second, the scheme uses an exact formula for the direct solution. Third, it does not demand a priori information about the subsurface resistivity distribution nor high computational resources. To our best knowledge, the R-parameter imaging method presented in this paper for the interpretation of self-potential data measured along profile by idealized models was not developed before. It is relevant to note that rigorous 3D inversion of self-potential data is computationally expensive, and requires a priori information for the model parameters (3D electric conductivity distribution) we invert for^17^.

The paper is structurally described as follows. The “Self-potential direct solution” section presents the direct problem (forward modeling solution). In “The method” section, the formulation of the proposed imaging scheme is explained. The “Numerical examples” section validates the method using synthetic models contaminated with a wide range of noise, regional self-potential signatures, and interference anomalies. Real data examples are carefully analyzed and discussed in the “Field examples” section, and finally some findings are reported.

**Figure 7:**
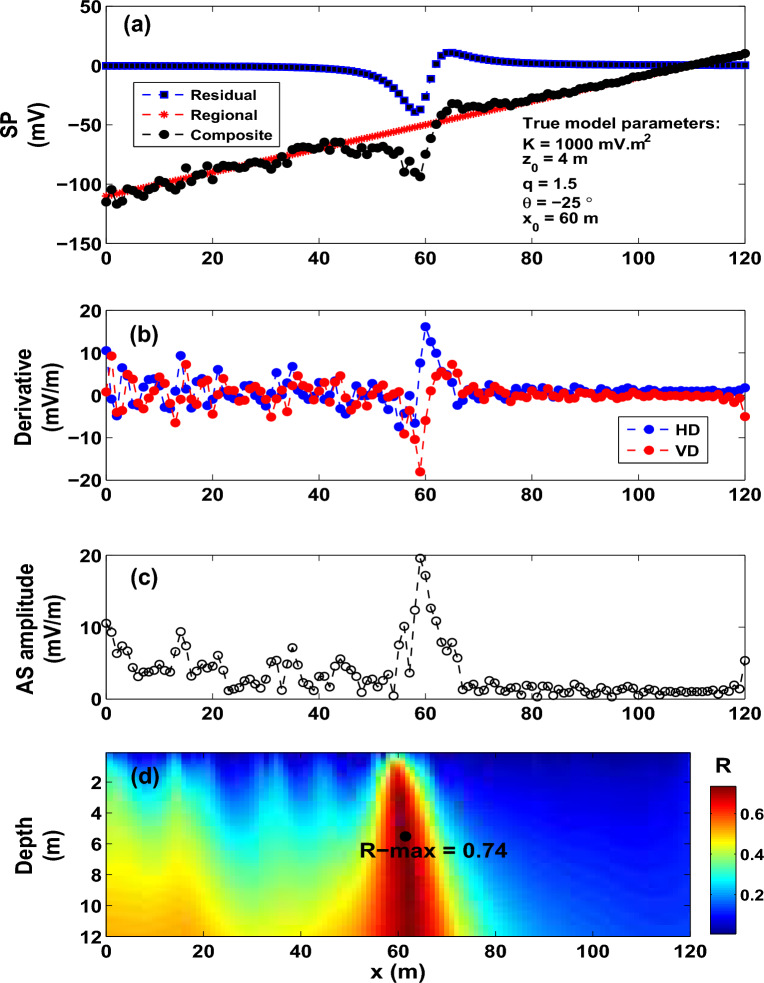
Model 4: noisy composite SP anomaly. (**a**) SP anomaly (generated by the SP data rendered in Fig. 6a) with 20% random noise. (**b**) Corresponding derivatives. (**c**) Amplitude. (**d**) Image.

**Figure 8:**
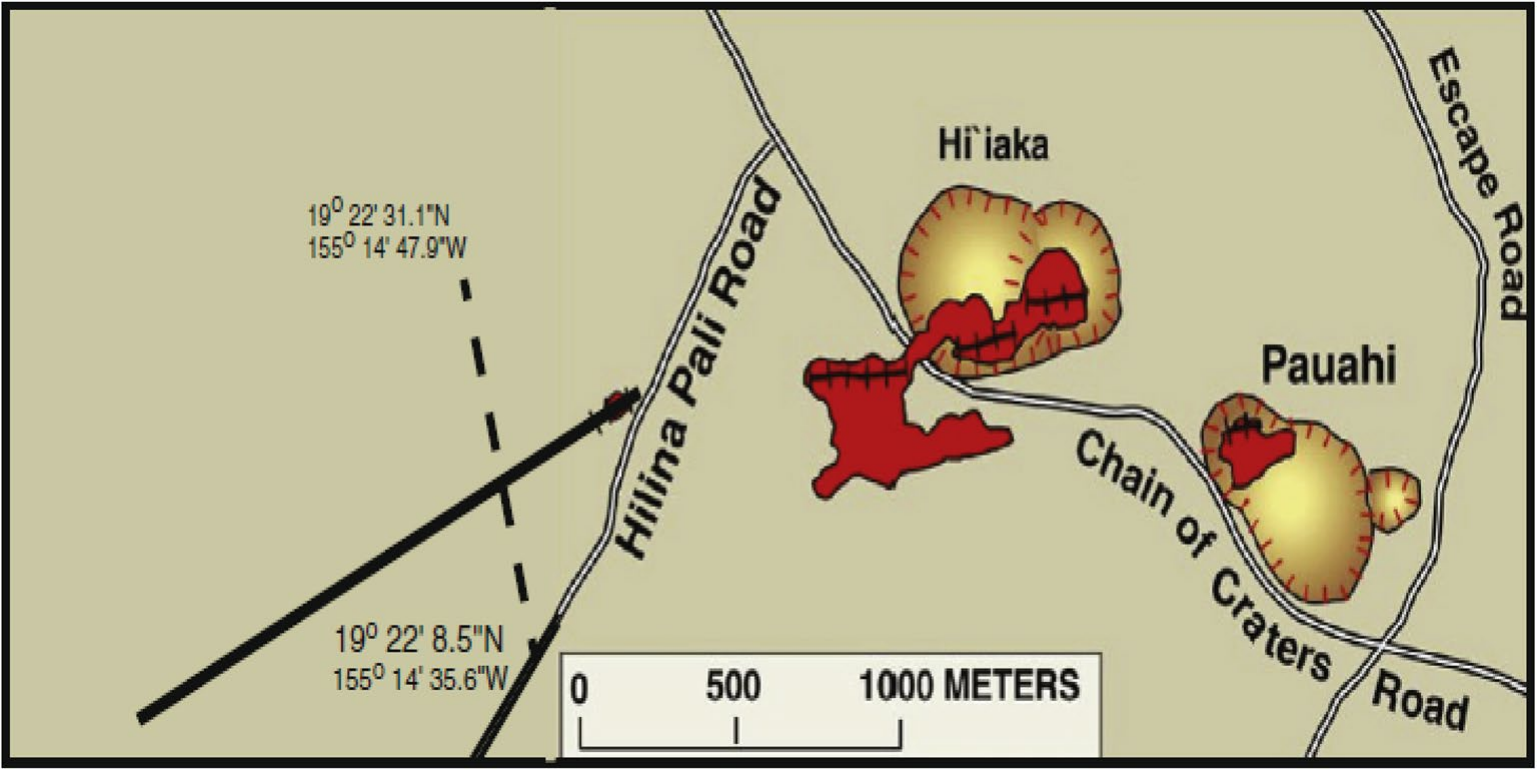
The Hi’iaka self-potential anomaly, the Kilauea volcano, Hawaii, USA. Profile of the self-potential measurements (dashed line). Inferred location of the Hi’iaka dike (solid line) (taken from Davis^83^ with permission from Elsevier).

## Self-potential direct solution

The self-potential signature (V) of some simple geometrical sources at an observation point ($x_j$, z) along profile (Fig. 1) is given by, e.g., Yungul^53^ and Mehanee^66^:

1 $$\begin{aligned} V(x_j,x_\circ , z,z_\circ , q,\theta ,K) = K \; \frac{(x_j-x_\circ )\cos (\theta )+ (z_\circ -z)\sin (\theta )}{ \Big [ (x_j - x_\circ )^{2} + (z_\circ -z)^{2} \Big ]^q}, \, \hspace{0.25 cm} j = 1, 2, 3,..., n \end{aligned}$$

where $x_j$ (m) is the coordinate of the measurement station (Fig. 1), *j* is the index of the measurement station, $x_{\circ }$ (m) is the origin point of the self-potential profile, $z_\circ$ and *z* (m) are the coordinates of the buried body and the observation station, $\theta$ (degrees) is the polarization angle, q (dimensionless) is the shape factor (*q* = 1.5, 1 and 0.5 for sphere, horizontal cylinder and semi-infinite vertical cylinder), *n* is the number of data points and *K* is the electric dipole moment. It is noted that the unit of *K* (mV m$^{2q-1}$) is function of the shape factor (*q*)^62^, and that $\theta$ is measured clockwise (Fig. 1) and ranges from 0 to − 180$^{\circ }$ in the above formula^8^.

**Table 2:** The Hi’iaka self-potential anomaly, the Kilauea volcano, Hawaii, USA. R-max versus q. See the text for details.

q	R-max			
(q)	1973	1995	1997	2012
0.5	0.9542	0.9475	0.9619	0.9689
**0.6**	0.9744	0.9671	0.9726	**0.9753**
**0.7**	0.9839	0.9757	**0.9754**	0.9718
**0.8**	**0.9858**	**0.9769**	0.9687	0.9667
0.9	0.9853	0.9761	0.9622	0.9648
1.0	0.9842	0.9745	0.9565	0.9627
1.1	0.9819	0.9704	0.9492	0.9587
1.2	0.9767	0.9635	0.9395	0.9535
1.3	0.9704	0.9559	0.9306	0.9474
1.4	0.9639	0.9484	0.9218	0.941
1.5	0.9575	0.9412	0.9135	0.9348

## The method

The AS expression^70^ is:

2 $$\begin{aligned} A_{s}(x_{j},z) = \frac{\partial V}{\partial x_{j}} - i \frac{\partial V}{\partial z}, \, \, \, \, i = \sqrt{-1} \end{aligned}$$

where $\frac{\partial V}{\partial z}$ and $\frac{\partial V}{\partial x_{j}}$ are the vertical and horizontal derivatives of the self-potential anomaly.

The analytic signal’s amplitude ($|A_{s}(x_j,z)|$) of the self-potential anomaly is given by Nabighian^70^:

3 $$\begin{aligned} \Big |A_{s}(x_{j},z)\Big |= \sqrt{\Big ( \frac{\partial V}{\partial x_{j}}\Big )^2+ \Big ( \frac{\partial V}{\partial z}\Big )_{}^2}. \end{aligned}$$

**Table 3:** The Hi’iaka self-potential anomaly, the Kilauea volcano, Hawaii, USA. Comparison.

Model	Davis^83^				Present study			
Parameters	1973	1995	1997	2012	1973	1995	1997	2012
$K ({mV\,m}^{2q-1})$	–	–	–	–	− **10688**	− **10072**	− **4340**	− **1718**
$z_{o}$ (m)	33 ± 3.2	17 ± 2.6	48 ± 3.3	170 ± 25	**54**	**57**	**69**	**177**
$x_{o}$ (m)	–	–	–	–	**300**	**280**	**310**	**340**
$\theta$ ($^{\circ }$)	–	–	–	–	− **90**	− **90**	− **110**	− **115**
*q*	–	–	–	–	**0.8**	**0.8**	**0.7**	**0.6**

**Table 4:** The Osnabr$\ddot{{u}}$ck self-potential anomaly, Germany. R-max versus q.

q	R-max
0.5	0.9931
0.6	0.9946
0.7	0.9938
0.8	0.9931
0.9	0.9948
1.0	0.9962
1.1	0.9972
**1.2**	**0.9976**
1.3	0.9974
1.4	0.9969
1.5	0.9959

Taking the vertical and horizontal derivatives of formula (1), and by substituting the results in expression (3), we get:

4 $$\begin{aligned} \scriptstyle \Big |A_{s}(x_{j},z)\Big |= |K| \; \frac{ \sqrt{((x_j-x_o)^2+ (z_\circ -z)^2)\Big [((x_j-x_o)^2+ (z_\circ -z)^2)+4q(q-1)\Big ((x_j-x_o)\cos (\theta )+ (z_\circ -z)\sin (\theta )\Big )^2\Big ]}}{\Big [(x_j-x_o)^2+ (z_\circ -z)^2\Big ]^{q+1}}. \end{aligned}$$

The R-parameter (correlation coefficient) is dependent upon both of the amplitude analytic signal of the actual self-potential data (*Aso*) observed along profile and that of the analytic signal amplitude of the calculated (theoretical) self-potential data (*Ast*) produced by an assumed source (for example a sphere):

5 $$\begin{aligned} R = \frac{\sum \limits _{j=1}^{n} [Aso]_{j} [Ast]_{j}}{\sqrt{\sum \limits _{j=1}^{n} {[Aso]_j}^2 \sum \limits _{j=1}^{n}{[Ast]_{j}}^2}}. \end{aligned}$$

The analytic signal [*Aso*] can be assessed numerically using expression (3), whereas the analytic signal [*Ast*] of an assumed source is calculated analytically from expression (4).

To calculate the parameters of a presumed source, the imaging parameter (R-parameter) is first plotted on a 2D map from which the depth $z_o$ can be readily read. It is worthy noting that when the parameters of the SP profile (Fig. 1) coincide with the buried anomalous source, the imaging parameter achieves its maximum value (so-called here R-max). Further pertinent detail is provided in the “Numerical examples” section. As is seen from expression (5), the computation of *R*($x_o$, $z_o$, $\theta$) does not need knowledge of the electric dipole moment *K*, which is calculated from the maximum self-potential response. Using expression (1), the predicted self-potential response is then calculated. Fig. 2 presents a flowchart showing the workflow of the developed scheme, which takes about 2 s on a simple PC to estimate the parameters of the interpretive model that resembles the buried anomaly.

**Figure 9:**
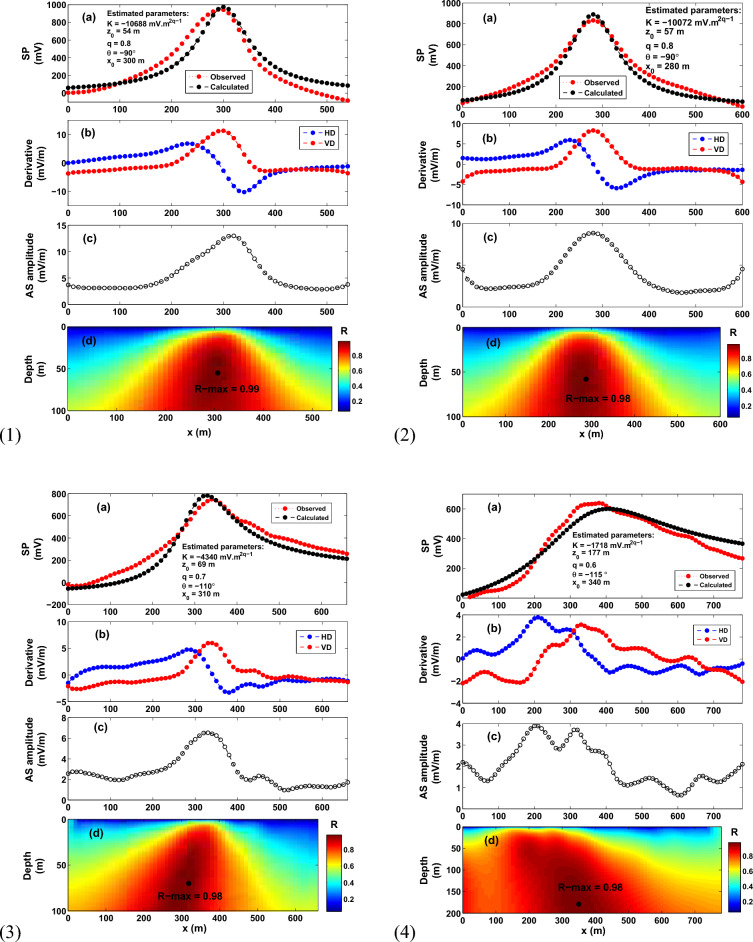
The Hi’iaka self-potential anomaly, the Kilauea volcano, Hawaii, USA. (1) The Hi’iaka SP anomaly profile surveyed in 1973. (2) The Hi’iaka SP anomaly profile surveyed in 1995. (3) The Hi’iaka SP anomaly profile surveyed in 1997. (4) The Hi’iaka SP anomaly profile surveyed in 2012. For each anomaly profile, (**a**) observed and calculated data. (**b**) Derivative of the observed SP data illustrated in (**a**). (**c**) Analytic signal amplitude. (**d**) Image.

**Figure 10:**
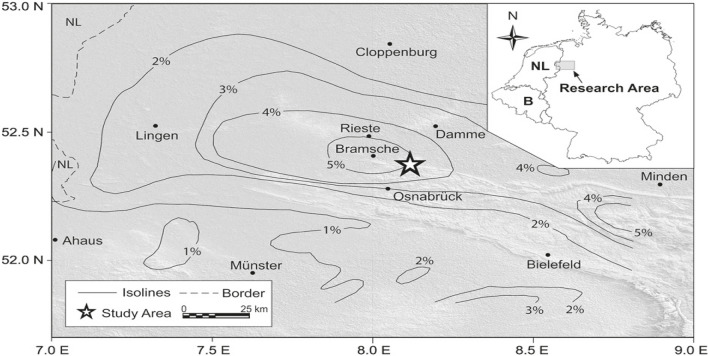
The Osnabr$\ddot{{u}}$ck self-potential anomaly, Germany. Location of the survey area (star) north of Osnbrück, and isolines of Vitrinite Reflectance of the maturity map (taken from Gurk et al.^6^ with permission from Elsevier). NL: Netherlands, B: Belgium.

## Numerical examples

The approach proposed here has been examined on synthetic self-potential data generated by various source models (for example, horizontal cylinder, sphere and vertical cylinder). The suggested scheme is first verified on numerical experiments without noise. After that, the data have been contaminated with realistic noise, and interpreted in order to assess the stability of the scheme e.g.^8,84^. Second, in order to assess the stability of the scheme further, the effect of the regional background (that is embedded into the measured self-potential data) on the results is carefully investigated.

**Figure 11:**
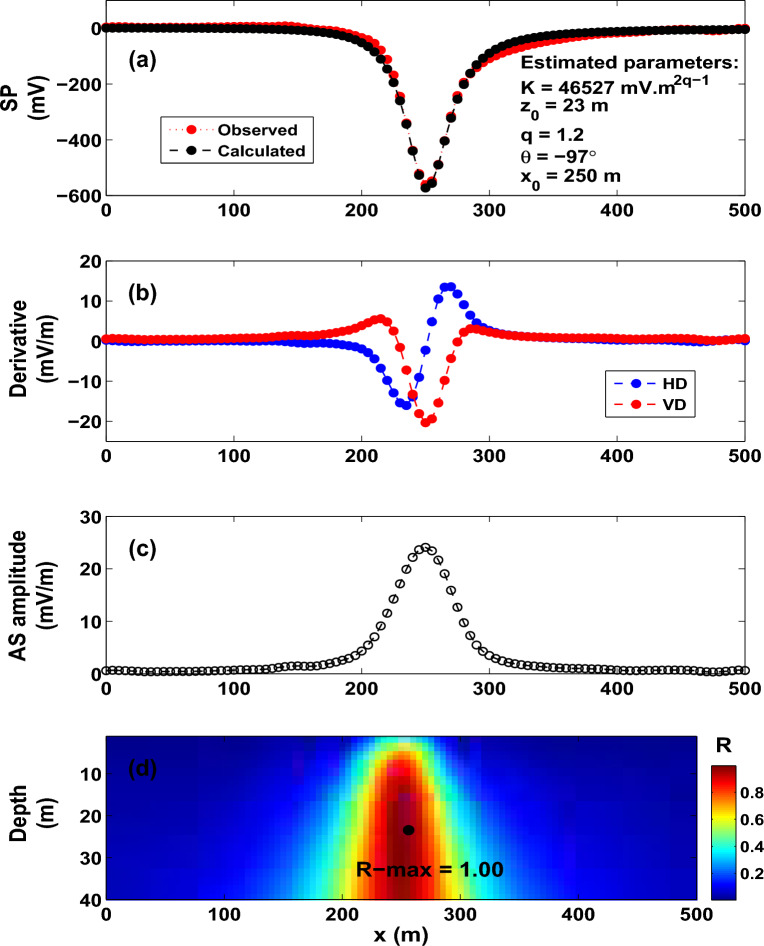
The Osnabr$\ddot{{u}}$ck self-potential anomaly, Germany. (**a**) Observed and calculated SP anomaly profile. (**b**) Derivatives of the observed SP anomaly. (**c**) Analytic signal amplitude. (**d**) Image (R-max = 0.9976 at *q* = 1.2, $\theta$ = $-97^{\circ }$, $z_o$ = 23 m and $x_o$ = 250 m).

### Model 1: horizontal cylinder

The self-potential response (Fig. 3a) of an idealized body of a horizontal cylinder shape (*K* = 2500 mV m, $z_o$ = 7 m, $\theta$ = $- 40^{\circ }$, $x_o$ = 60 m and profile length = 120 m) is calculated from formula (1). Following the recipe discussed above for the interpretation scheme proposed here, Fig. 3b renders the derivatives (horizontal and vertical) of the self-potential anomaly (Fig. 3a). The corresponding analytic signal amplitude (Fig. 3c) is then calculated from the spatial derivatives (Fig. 3b) using expression (3).

The mosaic surface S (which was gridded into 1-m spaces in the x- and z-directions) extended, respectively, to 120 $\times$ 12 m in these directions (that is ($x_{o}$, $z_o$) $\in$ S = (0, 120) $\times$ (1, 12)), and was used to compute and map the correlation coefficient (R-parameter). Expression (5) is employed to calculate the R-parameter for each possible sources (*q* = 0.5–1.5), where the largest value (R-max) of the R-parameter is attained at the true assumed source (that is q = 1 and R-max = 1.0) (Table 1). Figure 3d presents the image of the R-parameter composed using expression (5) assuming source of a horizontal cylinder model. The R-parameter’s maximum value is marked by the black dot, which denotes the true model parameters of the buried structure (Fig. 3d).

We reiterate that the R map (Fig. 3d) shows the 2D distribution of the obtained R-parameter values. The R-parameter measures the goodness of fit between the observed and predicted self-potential data, and is not representative of geologic structures. An R value of 1 means that the observed and predicted self-potential data are in perfect fit.

To further assess the developed imaging scheme, a number of shape values have been investigated. The scheme is found stable and can retrieve the true values of the model parameters as can be seen from the results presented in Fig. 4 and Table 1.

**Figure 12:**
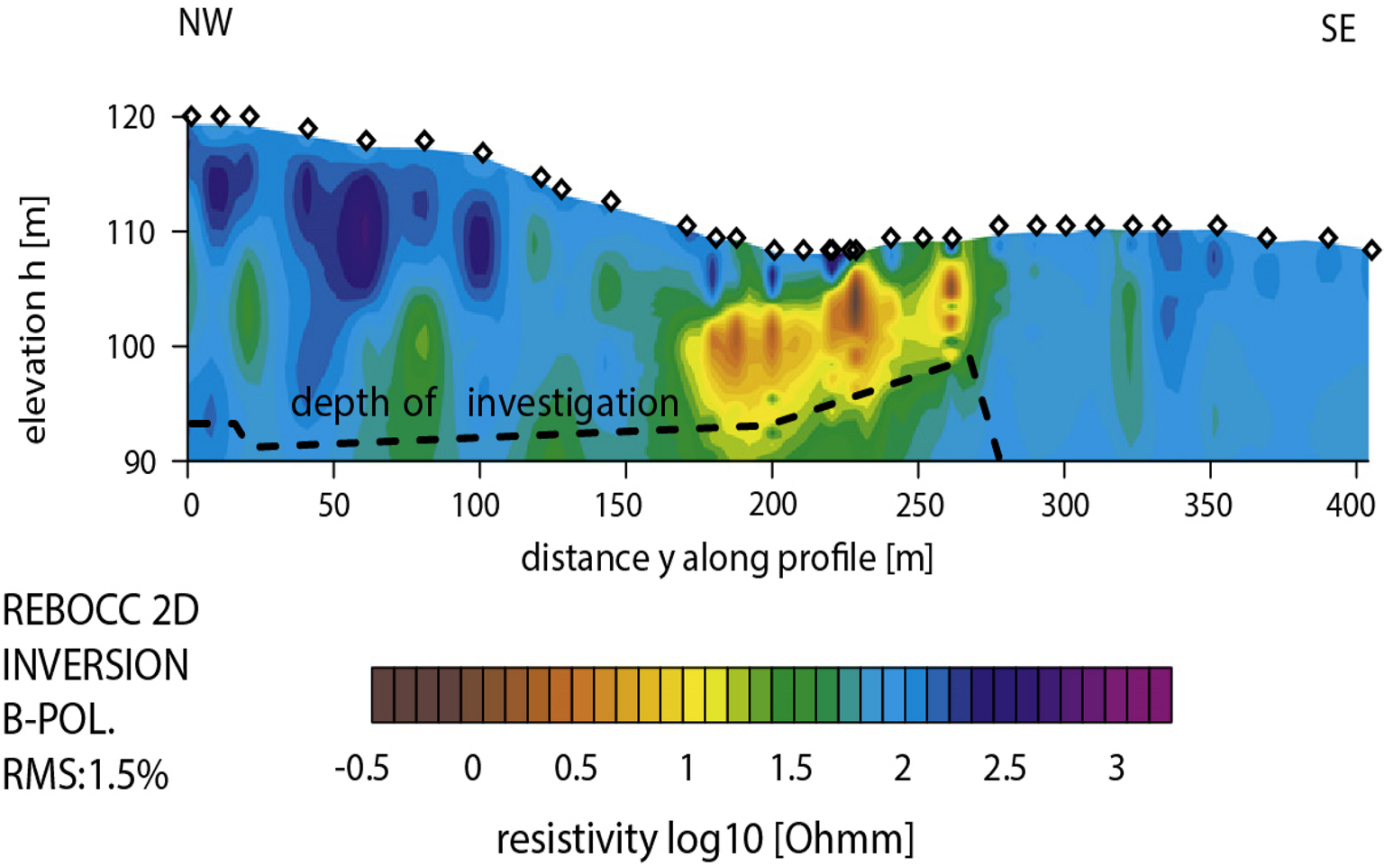
Osnabr$\ddot{{u}}$ck self-potential anomaly, Germany: Two-dimensional electromagnetic inversion results (taken from Gurk et al.^6^ with permission from Elsevier).

**Figure 13:**
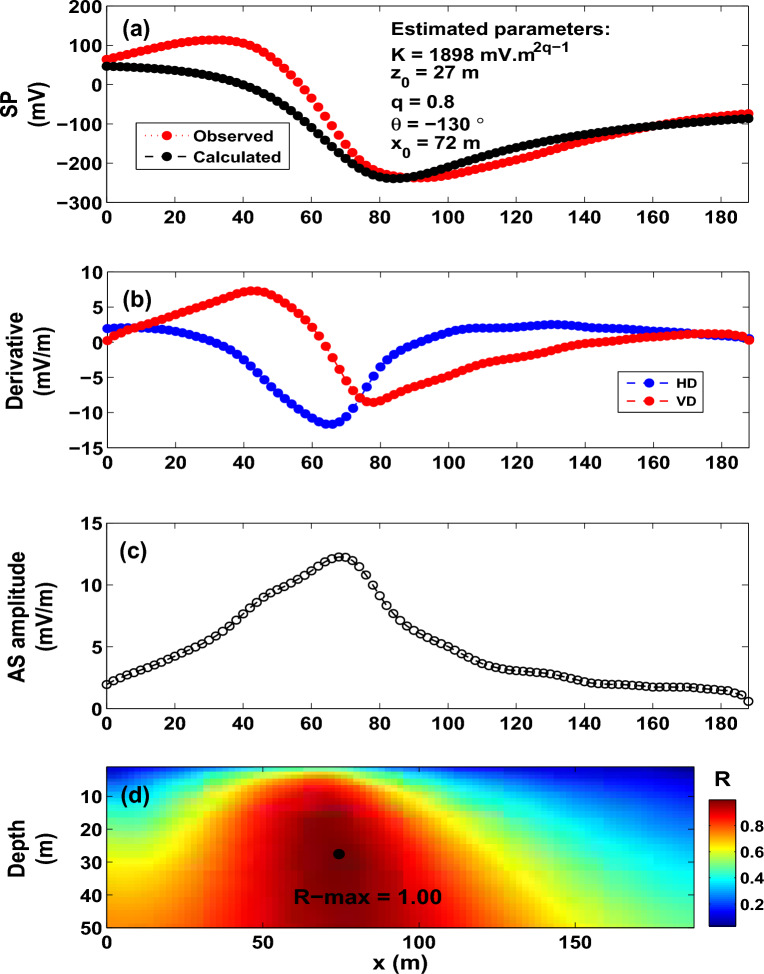
The Suleymankoy self-potential anomaly, Turkey. (**a**) Observed and calculated SP anomaly profile. (**b**) Derivatives of the observed SP anomaly. (**c**) Analytic signal amplitude. (**d**) Image (R-max = 0.9985 at *q* = 0.8, $\theta$ = $-130^{\circ }$, $z_o$ = 27 m and $x_o$ = 72 m).

We added 20% random noise into the self-potential data (Fig. 3a) of the above-mentioned synthetic model (Fig. 5a). Figure 5b,c show the spatial derivatives of the noisy self-potential anomalous signature (Fig. 5a) and their corresponding AS amplitude. Figure 5d shows the maximum R-parameter value (black dot) with a magnitude of 0.98. The estimated model parameters (*K*= 2906.30 mV m, $z_o$ = 7.4 m, $\theta$ = $- 42.60^{\circ }$, and $x_o$ = 60 m for an assumed shape factor *q* of 1.0) (Fig. 5d) are in good agreement with the actual ones.

It can be concluded from the above analysis that the R-parameter imaging method can produce accurate model parameters when the self-potential data are contaminated with noise.

**Figure 14:**
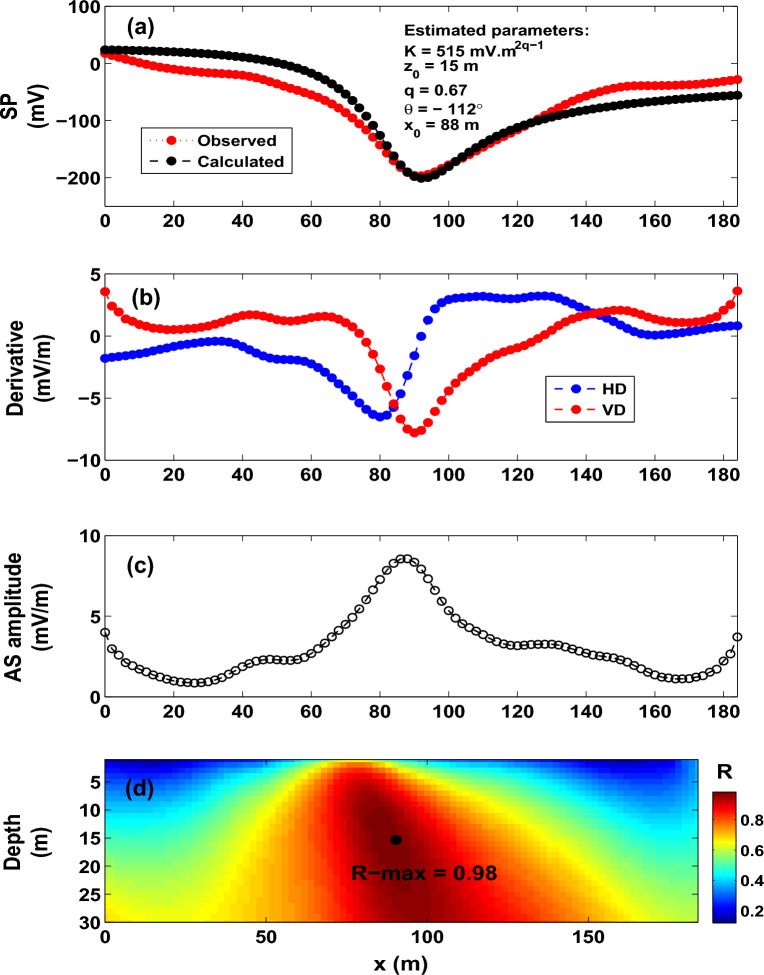
The Malachite Mine self-potential anomaly, USA. (**a**) Observed and calculated SP anomaly profile. (**b**) Derivatives of the observed SP anomaly. (**c**) Analytic signal amplitude. (**d**) Image (R-max = 0.9841 at *q* = 0.67, $\theta$ = $-112^{\circ }$, $z_o$ = 15 m and $x_o$ = 88 m).

### Model 2: sphere model

A synthetic self-potential anomaly for a sphere-shaped body (*K* = 1000 ${mV\,m}^2$, $z_o$ = 4 m, $\theta$ = $- 25^{\circ }$, $x_o$ = 60 m and profile length = 120 m) combined with a first-order regional anomaly generates the composite self-potential anomaly that is shown in Fig. 6a. The simulation formula of the composite anomaly has the form:

6 $$\begin{aligned} \Delta {V(x_j)} = \Big [1000 \; \frac{(x_j-60)\cos (-25)+ (4-0)\sin (-25)}{ [ (x_j - 60)^{2} + (4-0)^{2} ]^{1.5}}\Big ]+ (x_{j}-60) - 50 \end{aligned}$$

Following the procedures discussed above for Model 1, the derivatives of the composite SP response (Fig. 6a) are depicted in Fig. 6b. Figure 6c,d depict the corresponding analytic signal and distribution of the R-parameter values. Using a *q* of 1.5 (a sphere model), the pertinent model parameters (*K*=1155.6 mV m$^2$, $z_o$ = 4.3 m, $\theta$ = $- 25^{\circ }$, $x_o$ = 61 m) inferred from the imaging scheme are found in good agreement with the true values.

To assess the accuracy of the developed imaging scheme, the composite SP data profile (Fig. 6a) has been contaminated with 20% random noise (Fig. 7a). Figure 7b,c demonstrate the derivatives and the AS amplitude. The correlation map (Fig. 7d) rendered a maximum value of 0.74, which corresponds to an inverse model of *K*= 1640.60 mV m$^2$, $z_o$ = 5.4 m, $\theta$ = $-28^{\circ }$ and $x_o$ = 61 m for *q* = 1.5, which matches well the actual source model. This supports that the method has potential in exploration geophysics.

**Table 5:** The Osnabr$\ddot{{u}}$ck self-potential anomaly, Germany. Comparison.

Model Parameters	Gurk et al.^6^	Mehanee (2022)^8^	Present study
*K* (mV m$^{2q-1}$)	450 (mV)	11783.60 (mV m)	**46527** (mV m$^{1.4}$)
$z_{o}$ (m)	10–23	19.9	**23**
$x_{o}$ (m)	251.73	–	**250**
$\theta$ ($^{\circ }$)	95	− 99 .2	− **97**
*q*	thin sheet	1.0 (horizontal cylinder)	**1.2 (quasi horizontal cylinder)**

**Table 6:** The Suleymankoy self-potential anomaly, Ergani, Turkey. R-max versus q.

q	R-max
0.5	0.9762
0.6	0.9896
0.7	0.9977
**0.8**	**0.9985**
0.9	0.9973
1.0	0.9965
1.1	0.9958
1.2	0.9953
1.3	0.9950
1.4	0.9946
1.5	0.9940

## Field examples

The scheme is analyzed on five published real self-potential data from geothermal systems and mineral exploration in the following sections.

**Table 7:** The Suleymankoy self-potential anomaly data, Ergani, Turkey. Estimated parameters.

Model parameters	Yungul^53^	Essa et al.^17^	Srivastava and Agarwal^85^	Agarwal and Srivastava^86^	Biswas^23^	Present study
*K* (mV m$^{2q-1}$)	–	$-12072$	–	1560	10079.9	**1898**
$z_{o}$ (m)	38.8	35.9	28.9	27	27.8	**27**
$x_{o}$ (m)	–	–	64.1	68	72.2	**72**
$\theta$ ($^{\circ }$)	11	17.8	–	165	25.4	**-130**
*q*	–	1.0	1.0	1.0	1.0	**0.8**

### **The Hi’iaka self-potential anomaly, the Kilauea volcano, Hawaii, the United States of America**

A number of self-potential geophysical surveys were carried out in 1973, 1995, 1997 and 2012 over a basaltic dike intrusion (referred to as the Hi’iaka dike, Hawaii) (Fig. 8). The Hi’iaka dike intruded into the upper part of the Kilauea volcano, which is associated with the Hi’iaka and Pauahi craters eruption alongside the Kilauea rift zone^87,88^. It made a 100-m long surface fracture that erupted magma southwest of the Hi’iaka crater. Measurements of geophysical surveying recommended that the fracture is continued about 1.5 km under the subsurface in the southwest direction (Fig. 8).

The measurements of the self-potential data over the Hi’iaka dike intrusion commenced in 1973 by Zablocki^89^ and Zablocki^90^, and continued in 1995, 1997 and 2012^83^. Localized fluid disruption is blamed for the SP anomaly^83,89,90^. Davis^83^ stated that “*geothermal reservoirs are found above intrusions of magma such as dikes or dike swarms, which set up hydrothermal circulation generating hot water and steam from which energy can be tapped*”. The self-potential anomaly profiles of 1973, 1995, 1997, and 2012 are digitized into 10-m intervals (Fig. 9(1)–(4)). The Hi’iaka SP anomaly profiles have been interpreted by Davis^83^ using the self-potential inversion approach of Sill^38^. Davis^83^ interpreted the profiles by a trapezoidal source (approximated by a dike shaped model) located at different depths ranging from 50 to 190 m, and attributed the increase in depth to the magma cooling and heat loss at the top of the dike.

The aforementioned Hi’iaka self-potential anomaly profiles of 1973, 1995, 1997 and 2012 are interpreted using the scheme developed here (Fig. 9(1)–(4)). The derivatives of the self-potential response of each profile (Fig. 9(1)a,(2)a,(3)a,(4)a), and the corresponding analytic signal amplitude are rendered in Fig. 9(1)b,(2)b,(3)b,(4)b, and in Fig. 9(1)c,(2)c,(3)c,(4)c. The R-parameter values are reported in Fig. 9(1)d,(2)d,(3)d,(4)d with the R-max value for each SP profile. For the 1973, 1995, 1997, 2012 profiles, the estimated model parameters are found (*K* = -10688 mV m$^{{2q-1}}$, $z_o$ = 54 m, $\theta$= − 90, $x_o$ = 300 m, and *q* = 0.8 with an *R*-*max* of 0.99), (*K* = − 10072 mV m$^{{2q-1}}$, $z_o$ = 57 m, $\theta$= − 90, $x_o$ = 280 m, and *q* = 0.8 with an *R*-*max* of 0.98), (*K* = − 4340 mV m$^{{2q-1}}$, $z_o$ = 69 m, $\theta$= − 110, $x_o$= 310 m, and *q* = 0.7, with an *R*-*max* of 0.98), and (*K* = − 1718 mV m$^{{2q-1}}$, $z_o$ = 177 m, $\theta$= -115, $x_o$ = 340 m, and *q* = 0.6 with an *R*-*max* of 0.98). Table 2 tabulates the recovered model parameters for each profile, and shows that the observed self-potential anomaly is fit by a dike-like model with a shape factor of 0.6–0.8 (that is q = 0.6–0.8). Analysis shows that there is good match between the depths of the interpretive source (trapezoidal model) stated in the published literature and the depths obtained here (Table 3). The match between the observed and calculated self-potential data for each profile is depicted in Fig.9(1)a–(4)a, which is quite good.

It is re-noted that Davis^83^ reported that the SP anomaly remained strong throughout the measurement duration. However, the SP anomaly of 2012 is about 60% of that of 1973. Therefore, the variation in the recovered depths (54–177 m, Table 3) of the interpretated self-potential profiles (measured in 1973–2012) is not unexpected, and is attribuated to the magma cooling and heat loss at the top of the dike^83^.

**Figure 15:**
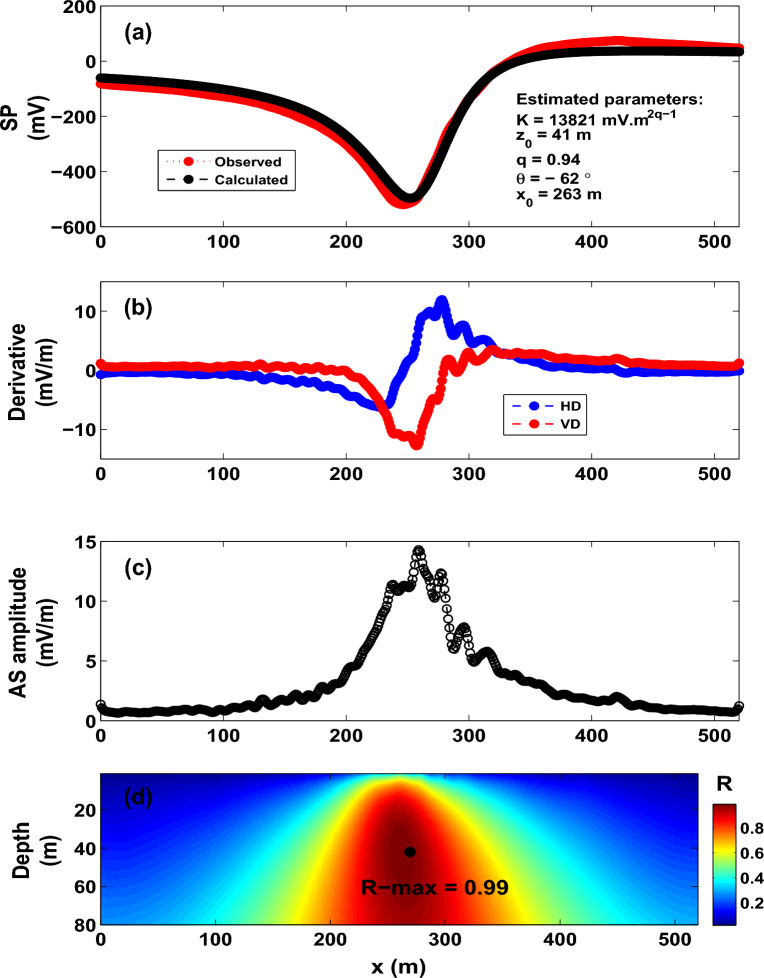
The Bavarian woods self-potential anomaly, Germany. (**a**) Observed and calculated SP anomaly profile. (**b**) Derivatives of the observed SP anomaly. (**c**) Analytic signal amplitude. (**d**) Image (R-max = 0.99 at *q* = 0.94, $\theta$ = $- 62^{\circ }$, $z_o$ = 41 m and $x_o$ = 263 m).

### **The Osnabr**$\ddot{{u}}$**ck self-potential anomaly, Germany**

A self-potential anomaly near the Osnabr$\ddot{{u}}$ck area (Fig. 10), Northwest Germany^6^ has been carried out to trace a graphite anomaly that has a quasi-vertical form in the Lias-epsilon shales. Gurk et al.^6^ found a significant single self-potential anomaly of roughly − 600 mV (Fig. 11a), which supports conductive graphite minerals. The 500-m long self-potential anomaly profile is meshed into 5-m intervals (Fig. 11a).

Figure 11b and c present the derivatives, and the AS amplitude of the SP anomaly, respectively. The R-parameter maximum value (R-max = 0.9976, black dot, Fig. 11d) was determined with the corresponding best interpretive parameters: *K* = 46527 mV m$^{{2q-1}}$, $z_o$ = 23 m, $\theta$ = $-97^{\circ }$, $x_o$ = 250 m, and *q* = 1.2 (Fig. 11a,d and Table 4). According to the recovered R-parameters, the subsurface anomalous body is approximated by a horizontal cylinder like-structure with a horizontal location of 250 m and an estimated depth to the center of 23 m, which is in good agreement with the interpreted results of Gurk et al.^6^ and Mehanee^8^ (Table 5). The variation in the magnitude of the model parameter *K* is due to the inconsistent use of unit (Table 5) as the interpretive models are not quite identical; they range from thin sheet to quasi-horizontal cylinder.

In order to map the 2D electric conductivity (inverse of resistivity) distribution in the underground, Gurk et al.^6^ measured a radio magnetotelluric data (apparent resistivities and phase) profile on the initial 400 m of the self-potential profile described earlier. The corresponding 2D inverse results of Gurk et al.^6^ revealed a prominent conductive anomalous body (Fig. 12), the location and depth of which correlate well with the results inferred from the approach developed here (Fig. 11).

**Table 8:** The Malachite Mine self-potential anomaly, Colorado, USA. R-max versus q.

q	R-max
0.5	0.9692
**0.67**	**0.9841**
0.7	0.9835
0.8	0.9770
0.9	0.9706
1.0	0.9675
1.1	0.9649
1.2	0.9601
1.3	0.9541
1.4	0.9475
1.5	0.9409

### **The Suleymankoy self-potential anomaly, Turkey**

The SP anomaly of Suleymankoy^53^ was carried out for copper deposits. The mine is characterized by alpine ophiolite containing several copper deposits. The anomaly is gridded at intervals of 2 m long (Fig. 13a). The self-potential anomaly of Suleymankoy is interpreted using the presented R-parameter imaging technique. Figure 13b–d depict the corresponding derivatives, the AS amplitude, and the imaging, which reveals an R-max value of 0.9985. The parameters revealed from interpretation are *K* = 1898 mV m$^{{2q-1}}$, $z_o$ = 27 m, $\theta$ = $-130^{\circ }$, $x_o$ = 76 m and *q* = 0.8 (Fig. 13a–d and Table 6). The observed and calculated self-potential data are rendered in Fig. 13a. Table 7 presents a comparison between the obtained results and those mentioned in the published literature.

**Table 9:** The Malachite mine self-potential anomaly, Colorado, USA. Estimated parameters.

Model parameters	Huff^91^	Dobrin^92^	Tlas and Asfahani^93^	Abdelrahman et al.^94^	Fedi and Abbas^4^	Mehanee^66^	Present study
*K* (mV m$^{2q-1}$)	–	–	$-299.28$ mV	$-241$ mV	–	209.0 mV	**515** mV m$^{0.34}$
$z_{o}$ (m)	12	15	12.79	17.3	13.6	12	**15**
$x_{o}$ (m)	–	–	–	–	83	–	**88**
$\theta$ ($^{o}$)	–	–	79.98	80	–	-95	**-112**
*q*	–	–	0.5	0.5	0.5	0.5	**0.67**

As can be seen from Table 7, the reported depths are in reasonable agreement, whereas the electric dipole moments (*K*) encountered some variations, which could be attributed to the various approximations employed in the interpretation schemes used in this table and how the parameter *K* is calculated from these schemes.

### **The Malachite mine self-potential anomaly, The United States of America**

The Malachite Mine is an amphibolite belt bounded by schists and gneisses. The self-potential profile over the Malachite Mine is digitized at intervals equal to 2 m (Fig. 14a).

The Malachite SP anomaly is interpreted using the R-parameter imaging technique. Figure 14b,c render the gradients, and the AS amplitude. Upon applying the imaging technique, an R-max of 0.9841 (Fig. 14d) was obtained with the following interpretive parameters: *K* = 515 mV, $z_o$ = 15 m, $\theta$ = $-112^{\circ }$, $x_o$ = 88 m, and *q* = 0.67 (Fig. 14a,d and Table 8). The subsurface structure was approximated by a semi-infinite vertical cylindrical structure with a horizontal spatial location of 88 m. The estimated depth (15 m to the top of the structure) is in good match with the drilling information and previous interpreted works (Table 9).

Table 9 shows that the parameter *K* encountered some variation; this is attributed to two main reasons. First, the inconsistent use of unit as the interpretive models are not quite identical; they range from vertical cylinder to quasi-vertical cylinder. Second, the nature of the approximations employed in interpretation schemes reported in this table, and how the parameter *K* is calculated from these schemes.

**Table 10:** The Bavarian woods self-potential anomaly, Germany. R-max versus q.

q	R-max
0.5	0.9583
0.6	0.9783
0.7	0.9929
0.8	0.9978
**0.94**	**0.9983**
1.0	0.9979
1.1	0.9974
1.2	0.9974
1.3	0.9965
1.4	0.9941
1.5	0.9911

### **The Bavarian woods self-potential anomaly, Germany**

Figure 15a depicts the self-potential anomaly collected over a graphite ore body at the southern Bavarian woods, Germany^95^. The self-potential anomaly profile is digitized using a 1-m sampling interval. Several authors have interpreted this anomaly profile. Al-Garani^96^ interpreted the anomaly as a quasi-vertical cylinder using the neural network inversion with $z_o$ = 33 m (depth to the top). Mehanee^66^ analyzed this SP profile with a horizontal cylinder of a depth to the center $z_o$ of 46 m using a regularized inversion. Gokturkler and Balkaya^5^ described the anomaly by a horizontal cylinder model using a genetic algorithm ($z_o$ = 45.03 m; to the center), simulated annealing ($z_o$ = 47.59 m; to the center) and particle swarm optimization algorithm ($z_o$ = 47.59 m). Di Maio et al.^97^ fit the profile by a horizontal cylinder by applying a spectral analysis and tomographic approach with $z_o$ = 44.9 m (to the center).

**Table 11:** The Bavarian woods self-potential anomaly, Germany. Estimated parameters.

Model parameters	Essa et al.^17^	Essa^98^	Al-Garani^96^	Gokturkler and Balkaya^5^			Mehanee^66^	Di Maio et al.^97^	Present study
				GA	PSO	SA			
*K* (mV m$^{2q-1}$)	30608.7	27212.7	2095	21272.9	33343.8	26257.4	27105	25000	**17325**
$z_{o}$ (m)	47.7	46.59	33	45.03	47.59	44.99	46	44.9	**41**
$x_{o}$ (m)	–	–	–	268.79	269.88	269.17	–	265.91	**263**
$\theta$ ($^{\circ }$)	−51.2	− 59.04	− 66	− 51.29	− 48.60	− 49.98	− 57	− 59.52	− **62**
*q*	1.0	1 .0	0.7	1.0	1.0	1.0	1.0	1.0	**0.94**

We interpreted this self-potential anomaly profile using the R-parameter imaging technique. The analytic signal amplitude anomaly calculated from the horizontal and vertical derivatives (Fig. 15b) is presented in Figure 15c. The R-parameter values are rendered in Fig. 15d. An R-max of 0.9983 pertains to an interpretive model of *K* = 13821 mV m$^{{2q-1}}$, $z_o$ = 41 m, $\theta$ = $-62^{\circ }$, $x_o$ = 263 m, and *q* = 0.94 (Fig. 15a,d and Table 10). The presented analysis shows that the depth and shape (which resembles a quasi-horizontal cylinder model) are generally in good agreement with the aforementioned results but the results of Al-Garani^96^, who interpreted the data by a quasi-vertical cylinder (q = 0.7) (Table 11). We do not expect all interpretation methods to yield the same results as each method has its own assumptions and limitation.

## Discussion

As mentioned above, the R map shows the 2D distribution of the obtained R-parameter values. The R-parameter measures the goodness of fit between the observed self-potential data, and the theoretical self-potential data generated from the model parameters (*z*, *K*, $\theta$) of the interpretive idealized model. The R parameter value does not provide uncertainty estimate for the evolved model parameters.

The non-uniqueness is one of the most challenging issues in geophysical data interpretation e.g.^99^, where multiple approximative solutions can equally fit the observed data. Joint inversion could help minimize this issue and provide better understanding e.g.^84^. It is worthy noting that it is very rare to solely use/measure one data kind when it comes to a detailed geophysical prospecting program. In industry, multiple data sets are essential for comprehensive understanding and for maximizing the potential of the underlying exploration program. Usually multiple geophysical data along with geological information are used, inverted and interpreted in an integrated manner (the so-called joint interpretation) to hopefully recover and select a unique inverse model the data of which match the measured data sets, and that fits into the underlying geologic setting of the area under study. May be this is the best we can do in exploration geophysics in order to resolve the non-uniqueness issue of an inverse problem solution.

## Conclusions

A rapid imaging scheme has been developed for the interpretation of self-potential data. In about 2 s on a simple PC, the scheme can estimate the parameters of the interpretive model (which is in the context of sphere, horizontal cylinder or vertical cylinder) that resembles the buried structure. The developed scheme uses the amplitude of the AS of the self-potential data undergoing interpretation and the amplitude of the AS of the self-potential data calculated by the assumed interpretive model to construct the corresponding 2D image of the so-called R-parameter. The scheme attains the largest value of the R-parameter when the recovered parameters coincide with the actual ones. It is noted that the R-parameter is independent of the electric dipole moment (*K*). The analyzed numerical examples demonstrated the stability of the developed scheme, and that its accuracy can be affected by the nearby geological structures. The five field data examples (from geothermal systems and mineral prospecting) analyzed here show that the scheme is capable of producing good results that agree well with those reported in other published research. The developed imaging scheme can have some potential in geothermal investigation and reconnaissance studies.

## Data Availability

The self-potential computer code and the datasets used and/or analysed during the current study are available from the corresponding author on reasonable request.
